# Inhibition of breast cancer growth in vivo by antiangiogenesis gene therapy with adenovirus-mediated antisense-VEGF

**DOI:** 10.1054/bjoc.2000.1734

**Published:** 2001-05

**Authors:** S-A Im, J-S Kim, C Gomez-Manzano, J Fueyo, T-J Liu, M-S Cho, C-M Seong, S N Lee, Y-K Hong, W K A Yung

**Affiliations:** Departments of 1Internal Medicine and Pathology, Ewha Medical Research Center, Ewha Womans University; Departments of General Surgery and Neurosurgery, Catholic University of Korea, Seoul, Korea; Department of Neuro-Oncology, The University of Texas M.D. Anderson Cancer Center, Houston, TX, USA

**Keywords:** breast cancer, antiangiogenesis, gene therapy, adenovirus, VEGF

## Abstract

Increased expression of VEGF in several types of tumours has been shown to correlate with poor prognosis. We used a replication-deficient adenoviral vector containing antisense VEGF cDNA (Ad5CMV-αVEGF) to down-regulate VEGF expression and increase the efficiency of delivery of the antisense sequence in the human breast cancer cell line MDA231-MB. Transfection of these cells with Ad5CMV-αVEGF in vitro reduced secreted levels of VEGF protein without affecting cell growth. Moreover, injection of the Ad5CMV-αVEGF vector into intramammary xenografts of these cells established in nude mice inhibited tumour growth and reduced the amount of VEGF protein and the density of microvessels in those tumours relative to tumours treated with the control vector Ad5(dl312). Our results showed that antisense VEGF _165_ cDNA was efficiently delivered in vivo via an adenoviral vector and that this treatment significantly inhibited the growth of established experimental breast tumours. The Ad5CMV-αVEGF vector may be useful in targeting the tumour vasculature in the treatment of breast cancer. © 2001 Cancer Research Campaign http://www.bjcancer.com


British Journal of Cancer (2001) 84(9), 1252–1257
© 2001 Cancer Research Campaign
doi: 10.1054/ bjoc.2000.1734, available online at http://www.idealibrary.com on IDEAL®
http://www.bjcancer.com

### Inhibition of breast cancer growth in vivo by antiangiogenesis gene therapy with adenovirus-mediated antisense-VEGF

**S-A Im[1], J-S Kim[2], C Gomez-Manzano[3], J Fueyo[3], T-J Liu[3], M-S Cho[4], C-M Seong[1], SN Lee[1], Y-K Hong[5] and WKA Yung[3]**

Departments of [1]Internal Medicine and [4]Pathology, Ewha Medical Research Center, Ewha Womans University; Departments of [2]General Surgery and [5]Neurosurgery, Catholic University of Korea, Seoul, Korea; [3]Department of Neuro-Oncology, The University of Texas M.D. Anderson Cancer Center, Houston, TX, USA

**Summary** Increased expression of VEGF in several types of tumours has been shown to correlate with poor prognosis. We used a replication-deficient adenoviral vector containing antisense VEGF cDNA (Ad5CMV-αVEGF) to down-regulate VEGF expression and increase the efficiency of delivery of the antisense sequence in the human breast cancer cell line MDA231-MB. Transfection of these cells with Ad5CMV-αVEGF in vitro reduced secreted levels of VEGF protein without affecting cell growth. Moreover, injection of the Ad5CMV-αVEGF vector into intramammary xenografts of these cells established in nude mice inhibited tumour growth and reduced the amount of VEGF protein and the density of microvessels in those tumours relative to tumours treated with the control vector Ad5(dl312). Our results showed that antisense $VEGF_{165}$ cDNA was efficiently delivered in vivo via an adenoviral vector and that this treatment significantly inhibited the growth of established experimental breast tumours. The Ad5CMV-αVEGF vector may be useful in targeting the tumour vasculature in the treatment of breast cancer. © 2001 Cancer Research Campaign http://www.bjcancer.com

**Keywords**: breast cancer; antiangiogenesis; gene therapy; adenovirus; VEGF

The growth of solid tumours and the formation of metastases depend on the formation of new blood vessels (Folkman, 1994). Angiogenesis is a complex multistep process involving extracellular matrix remodelling, endothelial cell migration and proliferation, and capillary differentiation and anastomosis, all of which are regulated by angiogenic peptides (Blood and Zetter, 1990). Vascular density has been shown to be an independent prognostic marker in several types of human tumours, including breast carcinoma (Weidner et al, 1992; Im et al, 1997). Vascular endothelial growth factor (VEGF, also known as vascular permeability factor) is a strong endothelial cell mitogen that increases the permeability of microvessels (Dvorak et al, 1995). The effects of VEGF are mediated through 2 distinct high-affinity endothelial cell surface receptors, flt-1 and KDR/Flk-1, both of which are type III tyrosine kinase receptors (Shibuya, 1995). Increased expression of VEGF has been reported in several types of human tumours and has been shown to correlate with poor prognosis (Takahashi et al, 1995; Toi et al, 1995). In animal models, antisense VEGF and monoclonal antibody to VEGF inhibited VEGF expression and tumour growth (Kim et al, 1993; Asano et al, 1995; Cheng et al, 1996; Saleh et al, 1996).

Several reasons exist to suggest that gene therapy should be considered as a strategy to suppress the neovascularization of solid tumours. First, local, intratumoral gene therapy can reduce the risk of widespread antiangiogenesis resulting from systemic administration of an antiangiogenic agent. Second, gene transfer can lead to a local accumulation of the antiangiogenic protein. Third, current technology does not allow genes to be transferred to all of the target cells that are relevant to the growth of the tumour; however, antiangiogenic gene therapy does not require that genes be transferred to all target cells. Increasing knowledge of the molecular basis of cancer has yielded several potential therapies, but the clinical application of these methods has not been completely successful, in part because the in vitro models used for screening often do not duplicate in vivo conditions. To overcome this limitation, we developed an adenovirally mediated antisense-VEGF vector, Ad5CMV-αVEGF, that could be used to down-regulate VEGF secretion and thereby inhibit tumour growth in vivo. We previously found that transfecting the U87-MG glioma cell line with this vector down-regulated $VEGF_{165}$ mRNA and VEGF protein secretion compared with cells that had been transfected with Ad5(dl312) (Im et al, 1999). The purpose of the present study was to test whether treating MDA231-MB human breast cancer xenografts with the Ad5CMV-αVEGF vector would reduce the expression of VEGF protein and suppress tumour formation through antiangiogenesis mechanism in an experimental model of breast cancer.

*Received 2 December 1999*
*Revised 7 August 2000*
*Accepted 20 November 2000*

*Correspondence to:* S-A Im

### MATERIALS AND METHODS

### Construction and generation of the adenoviral vectors

To down-regulate endogenous VEGF expression and enhance the in vivo applicability of the antisense VEGF strategy, we constructed a replication-deficient recombinant adenoviral vector containing the cDNA for $VEGF_{165}$ in an antisense orientation (Figure 1) as described previously (Zhang et al, 1993). Briefly, the 574-bp VEGF cDNA was cloned by using the pCRII vector (Invitrogen, Carlsbad, CA) and sequenced by using the T7 promoter and M13 reverse primers. Then, the VEGF cDNA was extracted from the pCRII vector with HindIII and Not I restriction enzymes and inserted in an antisense orientation into the E1-deleted expression plasmid pXCL-cytomegalovirus (CMV) shuttle vector (a generous gift from Dr WW Zhang, Urogen Corp, San Diego, California, USA) between the CMV promoter and SV40 polyadenylation signal site to create the expression plasmid pXCL-CMV-αVEGF. This plasmid was cotransfected with plasmid PJM17 into the transformed human embryonic kidney cell line 293 (American Type Culture Collection, Manassas, VA) by the calcium phosphate method. Homologous recombination of the expression plasmid and pJM17 in 293 cells replaced the E1 region with the expression cassette from the expression plasmid. Then individual viral plaques were isolated, and plaques containing the human $VEGF_{165}$ cDNA were identified by polymerase chain reaction (PCR) and restriction enzyme digestion and then amplified in 293 cells. Ad5(dl312), an E1-deleted adenovirus type 5 carrying no exogenous gene, was used as an adenoviral control vector (Jones and Shenk, 1979). Ad5CMV-αVEGF, Ad5(dl312), and Ad5CMV-βgal were propagated in 293 cells and then purified on a cesium chloride gradient. The preparations were then dialysed and stored in dialysis buffer (10 $mmolL^{-1}$ Tris-HCl and 1 $mmol^{-1}$ $MgCl_2$, pH 7.4) with 10% glycerol at –80˚C. The titre of each viral stock was determined in 293 cells by plaque assay (Graham and Prevec, 1991).

### Infection conditions

Cell lines were infected as described previously (Gomez-Manzano et al, 1996). Briefly, cells were infected by diluting the viral stock to a specified concentration, adding the dilution to cell monolayers, and incubating the cells at 37˚C for 30 min with brief agitation every 5 min. After this, culture medium containing 10% serum was added, and the infected cells were returned to the 37˚C incubator. Control cells were infected with the Ad5(dl312) or mock-infected with culture medium. Ad5CMV-βgal, an adenovirus type 5-based vector that lacks E1 and contains the CMV promoter that drives the *Escherichia coli* LacZ gene coding for the β-galactosidase protein, was used to verify the efficiency of infection.

### Cell line and tissue culture conditions

The human breast cancer cell line MDA231-MB was obtained from the American Type Culture Collection. MDA231-MB cells were maintained in DMEM/F-12 medium (1:1, vol:vol) supplemented with 10% fetal bovine serum in a humidified atmosphere containing 5% $CO_2$ at 37˚C. The 293 cells were maintained in high-glucose DMEM with 10% heat-inactivated fetal bovine serum.

### Transduction efficiency

To verify the transduction efficiency of the Ad5 vector, we infected MDA231-MB cells ($10^5$ cells per well) with Ad5CMV-βgal at multiplicities of infection (MOI) (the ratio of the number of infectious virions to the number of susceptible cells) ranging from 25 to 200. 48 hours later, cells were fixed with 4% paraformaldehyde in a phosphate-buffered NaCl solution, and then stained with 1 mg $ml^{-1}$ 5-bromo-4-chloro-3-indolyl-β-D-galactopyranoside in 5 mM potassium ferricyanide, 5 mM potassium ferrocyanide, and 2 mM $MgCl_2$ in phosphate-buffered NaCl solution at 37˚C overnight. The efficiency of infection (the percentage of β-galactosidase-positive cells) was determined by the number of blue cells among 500 cells on triplicate dishes.

### Enzyme-linked immunosorbent assay (ELISA)

To quantify human secretory $VEGF_{165}$ in conditioned medium, we used ELISA according to the manufacturer's protocol (R & D Systems, Minneapolis, MN). To prepare the conditioned medium, cells were seeded overnight ($10^5$ cells per well) with medium containing 10% serum and then infected with either Ad5CMV-α VEGF or Ad5(dl312) at different MOI. Culture medium was used for the mock-infection condition. Triplicate dishes of cells subjected to each treatment were used. Conditioned media were processed 5 days after infection as follows. 30 hours before media collection, the cells were washed 3 times with 2 ml of serum-free medium and then incubated with 2 ml of serum-free medium per well for 6 h, after which the medium was aspirated and the cells were washed again with 2 ml of serum-free medium per well and incubated for 24 hours in 1 ml of medium containing 2% serum. The medium was collected in a tube containing 1 μl of 100 mM phenylmethylsulphonyl fluoride.

### Cell growth rate in vitro

MDA231-MB cells were seeded at a density of $10^4$ cells per well in six-well culture plates and allowed to adhere overnight. The next day, the cells were infected with 100 MOI of Ad5CMV-αVEGF or Ad5(dl312) or with culture medium. Triplicate dishes of each treatment were counted at regular intervals until the 12th day after infection.

### Ad5CMV-αVEGF treatment in vivo

Animal experiments were carried out in the animal facility of The University of Texas MD Anderson Cancer Center in accordance with institutional guidelines. For these experiments, athymic female nu/nu mice, 4–6 weeks of age, were acclimated and caged in groups of 5 or fewer. All mice were fed a diet of animal chow and water ad libitum. The animals were anaesthetized with methoxyflurane before all procedures and were observed until full recovery. To create xenograft tumours, MDA231-MB cells ($5 \times 10^6$ in 100 μl of serum-free medium) were injected s.c. into the mammary fat pads of the mice. Beginning 4 days after tumour-cell implantation, tumours were treated by intratumoral injection of $5 \times 10^8$ plaque-forming units (PFU) of either Ad5(dl312) or Ad5CMV-αVEGF (8 mice/group) every other day for a total of 4 times; 3 of these 4-dose treatment cycles were conducted at 4-week intervals. Tumour volume was calculated from weekly caliper measurements of the largest (a) and smallest (b) diameters of each tumour using the formula $a \times b^2 \times 0.4$ (Attia and Weiss, 1966).

### Immunohistochemical analysis and microvessel counting

At the end of the 130-day measurement period, the mice were killed

© 2001 Cancer Research Campaign 

with $CO_2$, and their tumours were excised and fixed in neutral-buffered formalin for routine histologic examination and immunohistochemical staining. Tumours were analysed with an anti-VEGF mAb (PharMingen, San Diego, CA) to see the expression of VEGF and with an anti-CD31 mouse mAb (Biogenix, San Ramon, CA) to count microvessels as follows. Each paraffin block was serially sectioned at 4 μm intervals, dewaxed, and heated in a microwave for 10 min to retrieve antigen. Endogenous peroxidase in the histologic sections was removed by incubation with 3% hydrogen peroxide in water for 15 min at room temperature. The primary antibodies were diluted 1:200 (VEGF) or 1:10 (anti-CD31) in phosphate-buffered saline with 0.5% bovine serum albumin, and the dilutions were added to the sections and incubated either for 1 h at room temperature (VEGF) or overnight (CD31). The bound antibodies were detected by the avidin-biotin-peroxidase method and visualized with 3-amino-9-ethylcarbazole (AEC). Cellular nuclei were counterstained blue with haematoxylin.

The areas containing the greatest numbers of microvessels or tumour 'hot spots' were identified by scanning the stained sections at low magnification (40 × and 100 ×) with a light microscope. Once these areas were identified, individual stained microvessels were point-counted at 200 × magnification with a square grid that corresponded to a field size of 0.68 $mm^2$. Large microvessels and any single brown-stained endothelial cells that were clearly separate from other microvessels were included in the microvessel count; branching structures were counted as 1 vessel unless there was a break in the continuity of the vessel, in which case it was counted as 2 distinct vessels. The mean number of microvessels from 3 fields was used for analysis.

### Statistical analysis

All values were expressed as means ± standard deviations. Unpaired *t*-tests were used to evaluate differences in tumour volumes in the different treatment groups. A *P* value of less than 0.05 was considered to be statistically significant.

### RESULTS

### Adenovirus-mediated gene transfer in MDA231-MB cells

We assessed the efficiency of gene transfer via the replication-deficient recombinant adenoviral vector Ad5CMV-αVEGF, which contains the cDNA for $VEGF_{165}$ in an antisense orientation (Figure 1), in the MDA231-MB breast cancer cell line by measuring reporter gene expression 48 h after cells were infected with Ad5CMV-βgal at different MOI. The transduction efficiency ranged from 8.3 ± 2.05% at 25 MOI to 89.4 ± 3.30% at 200 MOI (Figure 2).

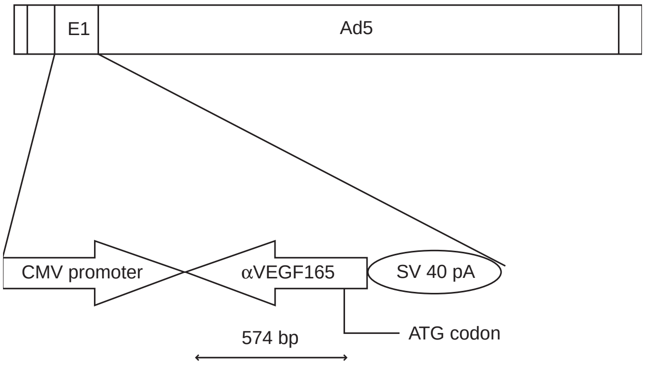

**Figure 1** Structure of the recombinant adenoviral vector Ad5CMV-αVEGF. The entire vector is shown at top, and an enlargement of human antisense-VEGF expression cassette is shown at bottom. The expression cassette contains human CMV promoter, human $VEGF_{165}$ cDNA (574 bp) in an antisense orientation, and the SV40 polyadenylation (pA) signal

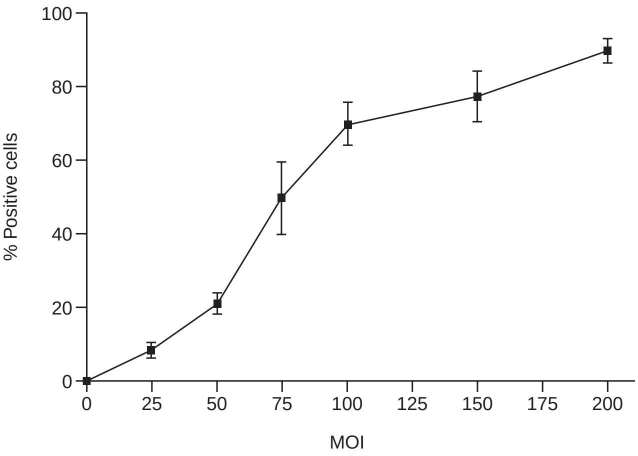

**Figure 2** Ad5CMV-βgal transduction efficiency in MDA231-MB breast cancer cells. Cells were infected with Ad5CMV-βgal at the MOI values shown. The percentage of blue cells were determined by counting the number of blue cells among 500 cells on each of 2 dishes, then dividing this number by 500 and multiplying by 100. The results shown are the means from triplicate experiments

### VEGF expression and cell growth after treatment with Ad5CMV-α VEGF in vitro

We used ELISA to determine the amount of secretory VEGF protein in conditioned medium collected 5 days after MDA231-MB cells has been mock-infected or infected with Ad5(dl312) or Ad5CMV-αVEGF. The mock-infected MDA231-MB cells secreted VEGF protein at a concentration of 110.98 ± 13.63 pg $ml^{-1}$ $10^5$ $cells^{-1}$ 24 $h^{-1}$; cells infected with Ad5(dl1312) produced 105.73 ± 10.03 pg $ml^{-1}$ $10^5$ $cells^{-1}$ 24 $h^{-1}$. In contrast, cells infected with 100 MOI of Ad5CMV-αVEGF produced 73.24 ± 7.02 pg $ml^{-1}$ $10^5$ $cells^{-1}$ 24 $h^{-1}$

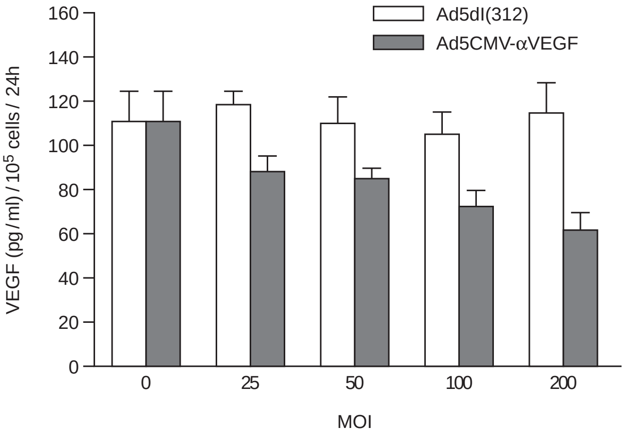

**Figure 3** VEGF protein production. MDA231-MB cells were infected with Ad5(d1312) or Ad5CMV-αVEGF, and conditioned medium collected 5 days later was subjected to ELISA. Error bars indicate SD from 3 replicate experiments

© 2001 Cancer Research Campaign

(Figure 3). Transfecting cells with 200 MOI of Ad5CMV-αVEGF reduced the amount of VEGF protein to 62.35 ± 8.24 pg $ml^{-1}$ $10^5$ $cells^{-1}$ 24 $h^{-1}$.

Despite the reduction in endogenous VEGF protein secretion by Ad5CMV-αVEGF-infected MDA231-MB cells, the growth rate of these cells was not different from that of mock-or Ad5(dl312)-infected MDA231-MB cells (Figure 4).

### Xenograft tumour growth after treatment with Ad5CMV-αVEGF

To determine the effectiveness of Ad5CMV-αVEGF therapy in vivo, we treated mice that had been implanted with MDA231-MB cells with periodic intratumoral injections of Ad5(dl312) or Ad5CMV-αVEGF (8 mice/group). 4 months after the treatment began (2 months after the final injection), the mean tumour size in the Ad5(dl312) treatment group was 335.23 ± 83.98 $mm^3$; mean tumour size in the Ad5CMV-αVEGF treatment group was 67.85 ± 34.65 $mm^3$ ($P = 0.0005$) (Figure 5). In addition to this direct evidence of an antitumor effect, Ad5CMV-αVEGF produced no adverse effects on gross observation of the health and behaviour of the mice.

### VEGF expression and microvessel count in tumours

Immunohistochemical analysis of tumour tissue that had been treated with Ad5(dl312) showed substantial VEGF protein expression in the diffuse pattern expected of a secreted protein. In contrast, little immunoreactivity was detected in the Ad5CMV-αVEGF-treated tumours (Figure 6). To determine whether the reduction in VEGF secretion and the inhibition of tumour growth were associated with a reduction in the ability of the cells to induce neovascularization, we assessed blood vessel density in tumour tissue by immunostaining the specimens with a monoclonal antibody against the endothelial cell surface protein marker CD31. Fewer microvessels were found in tumours treated with Ad5CMV-αVEGF (12.9 ± 1.85 per field) than in tumours treated with Ad5(dl312) (41.0 ± 6.72 per field) ($P = 0.03$).

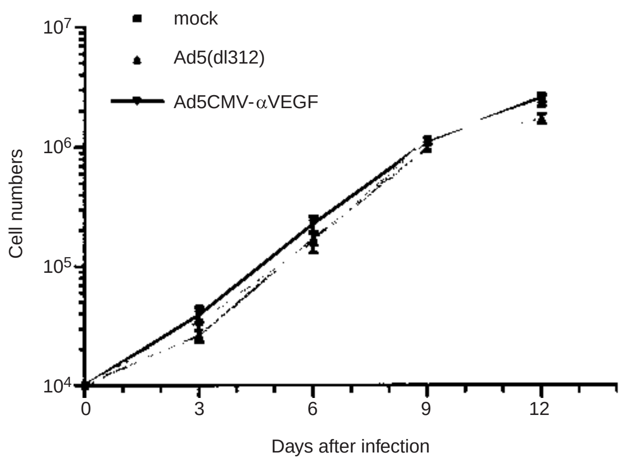

**Figure 4** Growth curves for MDA231-MB cells that were mock-infected or infected with 100 MOI of Ad5(d1312) or Ad5CMV-α VEGF. Cells were seeded at a density of $10^4$ cells per well and allowed to grow under standard culture conditions for 12 days. The cells were counted using a haemocytometer every 3 days. The numbers shown are means ± SD of triplicate dishes

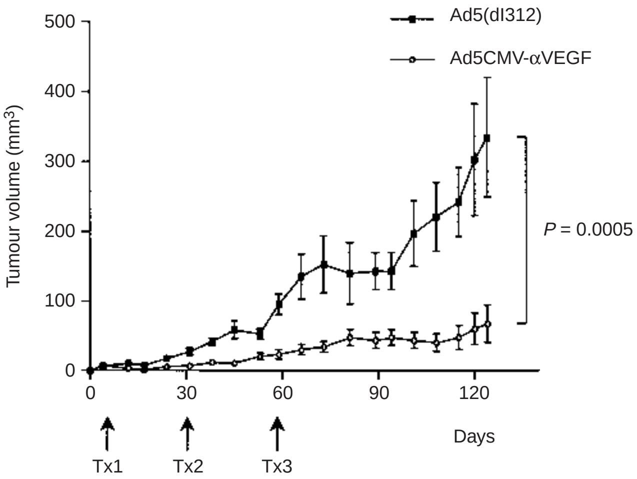

**Figure 5** Growth rates of subcutaneous tumours in nude mice treated with intratumoral injections of 5 × $10^8$ PFU Ad5(dl312) ($n = 8$) or Ad5CMV-αVEGF ($n = 8$). Injections were given once every other day over 8-day periods every 4 weeks starting 4 days after tumour-cell implantation. Tumour volumes were calculated from caliper measurements and are presented as the mean ± SEM ($mm^3$) for each group of mice

### DISCUSSION

The purpose of this study was to describe the in vitro and in vivo effect of Ad5CMV-αVEGF, a replication-deficient recombinant adenoviral vector carrying antisense human $VEGF_{165}$, in breast cancer. Our results showed that the transfer of antisense VEGF cDNA in vitro efficiently down-regulated the secretion of VEGF protein, both in vitro and in an experimental model of breast cancer.

VEGF is a potent endothelial cell mitogen and positive effector of angiogenesis. VEGF is known to be up-regulated in many tumour types (Kim et al, 1993; Asano et al, 1995; Cheng et al, 1996; Saleh et al, 1996; Im et al, 1997). In one study, VEGF RNA expression was significantly elevated in breast cancer specimens relative to that in samples of adjacent non-neoplastic tissue, whereas expression of bFGF, TGF-α and TGF-β RNA in tumour tissues was variable (Yoshiji et al, 1996). Up-regulation of VEGF in breast tumour specimens has been correlated with poor prognosis (Weidner et al, 1992; Toi et al, 1995). VEGF was shown in 1 study to be critical for the initial subcutaneous tumour growth of T-47D breast carcinoma cells, whereas other angiogenic factors could compensate for the loss of VEGF after the tumours reached a certain size (Yoshiji et al, 1997).

Our results, and those of others, suggest that targeting the endothelial cells of a tumour with antiangiogenic molecules can be an effective antitumor strategy. In one study, transfecting VEGF cDNA into xenografted C6 rat glioma cells produced hypervascularization of the tumour with abnormally large vessels; the abrupt withdrawal of VEGF resulted in regression of these vessels (Saleh et al, 1996). On the other hand, treating tumours with monoclonal antibodies specific to VEGF decreased or completely inhibited their neovascularization (Kim et al, 1993). Inhibition of endogenous VEGF expression by using an antisense VEGF sequence reduced vascularization and drastically suppressed the tumorigenicity of cancer cell lines (Saleh et al, 1996). We previously found that VEGF could be down-regulated in glioma cells by using ribozymes designed to target the VEGF sequence (Ke et al, 1998). In the present study, Ad5CMV-αVEGF did not affect the growth of the

*© 2001 Cancer Research Campaign*

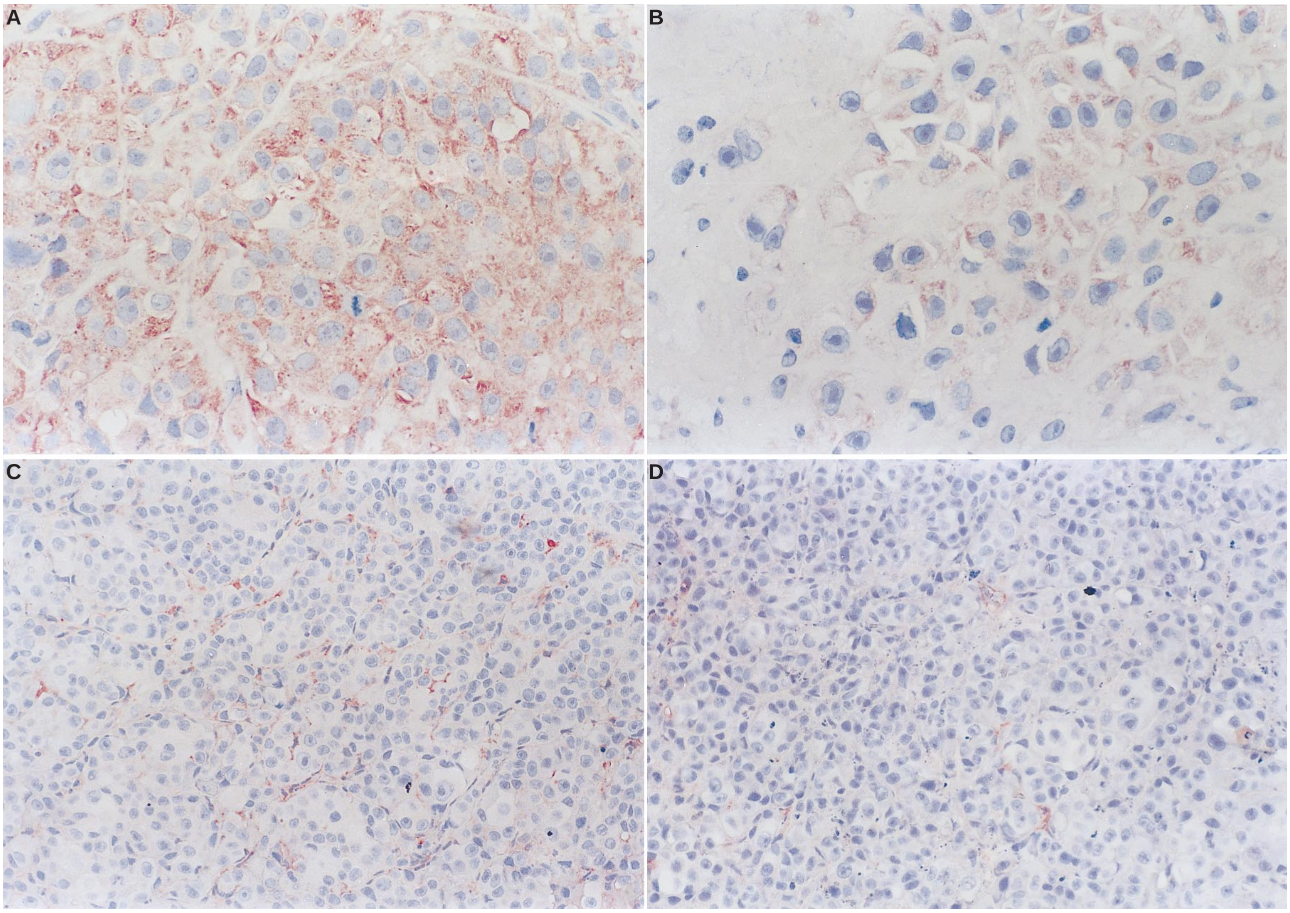

**Figure 6** Immunohistochemical staining of mammary fat pad xenografts of MDA231-MB cells with anti-VEGF antibody (**A,B**) or CD31 monoclonal antibody (**C,D**) after treatment with Ad5(d1312) (**A,C**) or Ad5CMV- αVEGF (**B,D**). VEGF expression, indicated by the intensity of brownish granules in the cytoplasm, was lower in the Ad5CMV-αVEGF-treated tumour (**B**) than in the Ad5(d1312)-treated tumour (**A**) (400×). Similarly, the number of brown-staining microvessels were much less dense in the Ad5CMV-αVEGF-treated tumour (**D**) than in the Ad5(d1312)-treated tumour (**C**) (200×)

tumour cells in vitro, but significantly suppressed the growth of tumour xenografts and reduced the levels of VEGF protein in vivo, a finding consistent with the apparent anticancer effects of other angiogenesis inhibitors (Beohm et al, 1997; Tanaka et al, 1998).

The results of our work suggest that anti-sense therapy targeting VEGF may have clinical implications for breast cancer treatment. However, several issues remain to be addressed, and some of them cannot be examined using currently available animal models. The host range of human adenoviruses is restricted, and no appropriate animal models exist to allow further exploration of the effectiveness of adenoviral vectors in immune-competent animal models. In a realistic scenario, intratumoral administration of the adenovirus in cancer patients, particularly after multiple treatments, may result in the production of neutralizing antibodies with subsequent elimination of infected cells. However, additional virus-induced cytotoxic effect might be beneficial. Thus, an immune response directed against viral antigens might augment tumour killing by affecting non-infected tumour cells. In addition, the intratumoral recruitment and stimulation of tumour-specific T lymphocytes theoretically could lead, in some cases, to systemic anti-tumor immunity.

In summary, we found adenovirus-mediated transfer of an anti-sense-VEGF gene to be an efficient means of delivering the gene to breast cancer cells in vitro and in vivo. Moreover, this successful delivery of the gene effectively decreased endogenous VEGF levels and suppressed the growth of tumours derived from human breast cancer MDA231-MB cells. These findings underscore the pivotal role of the VEGF system in breast tumour angiogenesis, and suggest that the Ad5CMV-αVEGF vector may be a useful tool for therapeutic targeting of the tumour vasculature in breast cancer.

### ACKNOWLEDGEMENTS

This study was supported in part by a grant from Ewha Womans University Medical College Graduates' Alumnae Association Research Fund, Korea and by US National Institutes of Health grants CA-51148 and CA-55261. We thank Polly S Lee and Mary T Wang for helpful discussion and purification of the viruses, Louis Hau and Christine Wogan for editorial assistance.

### REFERENCES

Asano M, Yukita A, Matsumoto T, Kondo S and Suzuki H (1995) Inhibition of tumor growth and metastasis by an immunoneutralizing monoclonal antibody to human vascular endothelial growth factor/vascular permeability factor 121. *Cancer Res* **55**: 5296–5301

 *© 2001 Cancer Research Campaign*

Attia MA and Weiss DW (1966) Immunology of spontaneous mammary carcinomas in mice. V: acquired tumor resistance and enhancement in strain of mice infected with mammary tumor virus. *Cancer Res* **26**: 1787–1800
Boehm T, Folkman J, Browder T and O'Reilly MS (1996) Antiangiogeneic therapy of experimental cancer does not induce acquired drug resistance. *Nature* **390**: 404–407
Blood CH and Zetter BR (1990) Tumor interactions with the vasculature: angiogenesis and tumor metastasis. *Biochem Biophys Acta* **1032**: 89–118
Cheng S, Su Huang H, Nagane M, Ji X, Wang D, Shih CC, Arap W, Huang C and Cavenee WK (1996) Suppression of glioblastoma angiogenicity and tumorigenicity by inhibition of endogeneous expression of vascular endothelial growth factor. *Proc Natl Acad Sci USA* **93**: 8502–8507
Dvorak HF, Detmar M, Claffey KP, Nagy JA, Van De Water L and Senger DR (1995) Vascular permeability factor/vascular endothelial growth factor: an important mediator of angiogenesis in malignancy and inflammation. *Int Arch Allergy Appl Immunol* **107**: 233–235
Folkman J (1994) Angiogenesis and breast cancer. *J Clin Oncol* **12**: 441–443
Gomez-Manzano C, Fueyo J, Kyritsis AP, Steck PA, Roth JA, McDonnell TJ, Steck KD, Levin VA and Yung WKA (1996) Adenovirus- mediated transfer of the p53 gene produces rapid and generalized death of human glioma cells via apoptosis. *Cancer Res* **56**: 694–699
Graham FL and Prevec L (1991) Manipulation of adenovirus vectors. In: Murray EJ (ed) *Gene Transfer and Expression Protocols* (Methods in Molecular Biology, Vol. 7), pp 109–128. Human Press, Inc: Clifton NJ
Im SA, Lee SN and Kim SS (1997) Tumor angiogenesis as a predictor of prognosis in gastric carcinoma. *J Korean Cancer Assoc* **29**: 640–647
Im SA, Gomez-Manzano C, Fueyo J, Liu TJ, Ke LD, Kim JS, Lee HY, Steck PA, Kyritsis AP and Yung WKA (1999) Antiangiogenesis treatment for gliomas: transfer of antisense-vascular endothelial growth factor inhibits tumor growth in vivo. *Cancer Res* **59**: 895–900
Jones N and Shenk T (1979) An adenovirus type 5 early gene function regulates expression of other early viral genes. *Proc Natl Acad Sci USA* **76**: 3665–3669
Ke LD, Fueyo J, Chen X, Steck PA, Shi YX, Im SA and Yung WKA (1998) A novel approach to glioma gene therapy: down-regulation of the vascular endothelial growth factor in glioma cells using ribozymes. *Int J Oncol* **12**: 1391–1396
Kim KJ, Li B, Winer J, Armanini M, Gillett N, Phillips HS and Ferrara N (1993) Inhibition of vascular endothelial growth factor-induced angiogenesis suppresses tumor growth in vivo. *Nature* **362**: 841–844
Saleh M, Stacker SA and Wilks AF (1996) Inhibition of growth of C6 glioma cells in vivo by expression of antisense vascular endothelial growth factor sequence. *Cancer Res* **56**: 393–340
Shibuya M (1995) Role of VEGF-Flt receptor system in normal and tumor angiogenesis. *Adv Cancer Res* **67**: 281–316
Takahashi Y, Kitadai Y, Bucana CD, Cleary KR and Ellis LM (1995) Expression of vascular endothelial growth factor and its receptor, KDR, correlates with vascularity, metastasis, and proliferation of human colon cancer. *Cancer Res* **55**: 3964–3968
Tanaka T, Cao Y, Folkman J and Fine HA (1998) Viral vector- targeted antigangiogenic gene therapy utilizing an angiostatin complementary DNA. *Cancer Res* **58**: 3362–3369
Toi M, Inada K, Suzuki H and Tominaga T (1995) Tumor angiogenesis in breast cancer: its importance as a prognostic indicator and the association with vascular endothelial growth factor expression. *Breast Cancer Res Treat* **36**: 193–204
Weidner N, Folkman J, Pozza F, Bevilacqua P, Allred EN, Moore DH, Meli S and Gasparini G (1992) Tumor angiogenesis: a new significant and independent prognostic indicator in early stage breast carcinoma. *J Natl Cancer Inst* **84**: 1875–1887
Yoshiji H, Gomez DE, Shibuya M and Thorgeirsson UP (1996) Expression of vascular endothelial growth factor, its receptor, and other angiogenic factor in human breast cancer. *Cancer Res* **56**: 2013–2016
Yoshiji H, Harris SR and Thorgeirsson UP (1997) Vascular endothelial growth factor is essential for initial but not continued in vivo growth of human breast carcinoma cells. *Cancer Res* **57**: 3924–3928
Zhang WW, Fang X, Branch CD, Mazur W, French BA and Roth JA (1993) Generation and identification of recombinant adenovirus by liposome-mediated transfection and PCR analysis. *Biotechniques* **15**: 868–872

*© 2001 Cancer Research Campaign*
